# CircRNA expression profiles of breast cancer and construction of a circRNA-miRNA-mRNA network

**DOI:** 10.1038/s41598-022-21877-y

**Published:** 2022-10-22

**Authors:** Liping Xu, Mengmeng Lyu, Sujin Yang, Jian Zhang, Dandan Yu

**Affiliations:** 1 Department of Radiation Oncology, The First Affiliated Hospital of Nanjing Medical University, Guangzhou Road 300, Nanjing, 210029 People’s Republic of China; 2 Department of Gynecologic Oncology, Jiangsu Cancer Hospital and Jiangsu Institute of Cancer Research and The Affiliated Cancer Hospital of Nanjing Medical University, Nanjing, People’s Republic of China; 3 Department of General Surgery, The First Affiliated Hospital of Nanjing Medical University, Nanjing, 210029 People’s Republic of China

**Keywords:** Cancer, Genetics, Oncology

## Abstract

CircRNAs are a group of endogenous small noncoding RNAs that are involved in multiple diseases including cancers. At present, the functions of circRNAs in breast cancer need to be further explored. In this study, 3 pairs of breast cancer and paracancer tissues with axillary lymph node metastasis were collected for circRNA high-throughput sequencing. We have identified 17,966 distinct circRNA candidates. Significant differential expressions were found in 136 circRNAs in breast cancer tissues relative to the matched paracancer tissues. We aslo identified differentially expressed 156 miRNAs and 1105 mRNAs in breast cancer tissues and normal breast tissues from public databases. Then we constructed a regulatory ceRNA network. 12 mRNAs were associated with prognosis of breast cancer. We also constructed a circRNAs-mediated subnetwork which might be related to prognosis of breast cancer. This article provides a better understanding of circRNAs-mediated ceRNA regulatory network by which circRNAs compete for endogenous RNAs in breast cancer.

## Introduction

Breast cancer is the most commonly occurring cancer and the dominant induction of cancer-associated death in females globally. There were about 2.3 million new cases and 685,000 deaths of breast cancer worldwide in 2020^1^. Breast cancer can be treated by various approaches, including surgery, radiotherapy, and chemotherapy. Molecular biomarkers of breast cancer include ER, PR, Her-2, EFGR and so on. Therapies targeting hormone and her-2 receptors become standard breast cancer treatments. These effective treatment advances in recent years have brought remarkable survival gains for breast cancer patients^2,3^. Nevertheless, the survival rate of distant-stage breast cancer is much lower than that of regional-stage one^4^. Distant metastasis and lymph node metastasis (LNM) will worsen the prognosis and survival of breast cancer patients. The progression of breast cancer metastasis is complicated and involves various molecules and pathways. Therefore, searching for new biomarkers of breast cancer metastasis may provide new treatments of metastatic breast cancer.

Circular RNAs (circRNA), a group of endogenous small noncoding RNAs, were identified in 1990s and initially thought to be useless products of RNA splicing^5^. With the development of high-throughput sequencing, an increasing number of functional circRNAs have been discovered. CircRNAs are characterized by closed loops formed by covalent bonds and are stable enough to resist degradation by RNA exonucleases^6,7^. Current knowledge shows that circRNAs function by three mechanisms: acting as competing endogenous RNAs (ceRNA), regulating gene transcription in the nucleus, and binding and coding proteins^8–12^. CircRNAs are attracting growing attention because of their multiple functions in various cancers. For example, circIRAK3 regulates malignant progression of breast cancer through sequestering miR-3607^9^, and circ-FBXW7 represses glioma tumorigenesis by encoding novel proteins^12^.

Currently, only a few studies have reported the functions of circRNAs in breast cancer. Herein, our team acquired the circRNA expression profile in breast cancer tissues with LNM and in matched paracancer tissues using high-throughput RNA sequencing. We selected 136 significantly differentially expressed circRNAs in breast cancer and paracancer tissues, which may be involved in the invasion and metastasis of breast cancer. We also collected the miRNA and mRNA expression profiles in breast cancer and paracancer tissues from the Gene Expression Omnibus (GEO) database. We identified the miRNAs and mRNAs with differential expressions and constructed a circRNA-miRNA-mRNA network. To further explore the role of circRNAs in breast cancer, we used the data from The Cancer Genome Atlas (TCGA, https://portal.gdc.cancer.gov/) to screen mRNAs associated with breast cancer prognosis, and constructed a circRNA-miRNA-mRNA subnetwork associated with breast cancer prognosis.

## Methods

### Sample information and collection

We collected 13 pairs of breast cancer and matched paracancer tissues from 13 patients who received axillary lymph node dissection in Jiangsu Province Hospital. We took one breast cancer tissue sample and one paracancer breast tissue from one breast cancer patient. Three pairs of axillary LNM samples were picked according to postoperative pathological information. The immunohistochemical and pathological details of these 3 pairs are listed in Table 1.

**Table 1:** Pathological and immunohistochemistry information of the 3 pairs of samples.

Sample	Gender	immunohistochemistry	Lymphatic invasion (negative/positive)	Pathological stage
1	Female	ER (70%+), PR (60%+), Her-2 (+), Ki67 (20%+)	11/20	pT2N3M0, IIIC
2	Female	ER (> 90%+), PR (75%+), Her-2 (-), Ki67 (25%)	8/33	pT1cN2M0, IIIA
3	Female	ER (95%+), PR (< 10%+), Her-2 (2+), Ki67 (20%)	1/17	pT2N1M0, IIB

Surgeons removed the breast tumors and adjacent normal tissues and immediately sent them to the pathology department. Pathologists excised a part of the tumors, and placed them and adjacent normal tissues separately into sealed tubes, which were then put in liquid nitrogen. This study was performed with approval from the Ethics Committee of the Hospital and with informed consent forms from all participants.

### Total RNA extraction and quality control

Total RNA from tissues was isolated with TRIzol agents (Invitrogen, US). Quality and purity of total RNA were detected by an Agilent 2100 instrument (Agilent Technologies, US) and a Nanodrop microspectrophotometer (Thermo Fisher Scientific, US).

### CircRNA sequencing

CircRNA sequencing was delegated to Gene Denovo Company (Guangzhou, China). Total RNA of each breast tissue sample was prepared for circRNA sequencing libraries. In brief, ribosomal RNAs (rRNA) and linear RNAs were removed from total RNA by a Ribo-Zero Gold rRNA removal kit (Illumina, US) and a Ribonuclease R kit (Epicentre, US) respectively. Double-strand cDNA was synthesized by PCR. The cDNA was subjected to adapter ligation, A-tailing and end restoring on a NEBNext poly(A) mRNA magnetic extraction module (New England Biolabs, US). Then 12 cycles of PCR amplification were carried out, followed by purification with AMPure XP beads (Beckman, US). Quality of sequencing libraries was controlled by a DNA 1000 assay kit (Agilent Technologies). The circRNA was sequenced on an Illumina NovaSeq 6000 system (Illumina).

Raw reads were obtained from sequencing machines, and the low-quality adapter reads were discarded to get clean reads, which were aligned to the rRNA database on Bowtie2^13^. After removal of rRNA mapped reads, the rest reads were mapped to the control genome on TopHat2 2.0.3.12^14^. Afterwards, the unmapped reads were selected for circRNA recognition.

### CircRNAs identification

From the two ends of the selected reads, 20mers were extracted and aligned to Ensembl release 88 GRCh38, forming unique anchor places within the splice site. Anchor reads aligned reversely (head-to-tail) implied circRNA splicing. Those anchor reads were submitted to find_circ software^15^, which identified 17,966 circRNAs. These circRNAs were mapped to the circBase on Blast. Highly reliable annotated circRNAs were found and defined as existing circRNAs, and others were regarded as new circRNAs. The circRNA expressions were quantified using the back-spliced Reads Per Million mapped Reads (RPM). We acquired the distribution of circRNAs for the compared samples through violin plot by R.

### Data acquisition

We downloaded miRNA and mRNA datasets (GSE143564 and GSE50428; analysed on Affymetrix Multispecies miRNA-4 Array and Agilent-021412 nONCOchip_1.0 021253) from GEO (https://www.ncbi.nlm.nih.gov/geo/). Then we transformed the probes to the matching gene symbols according to the annotation information. The GSE143564 dataset contained 3 breast cancer tissues and 3 adjacent tissues. The GSE50428 dataset contained 5 normal breast tissues and 26 breast cancer tissues. The TCGA database contains mRNA expression data, clinical information, and prognostic information for a variety of tumors. We obtained mRNA expression data of 1053 breast cancer samples and prognostic information from TCGA database.

### Identification of differentially expressed circRNAs, miRNAs and mRNAs

We used paired t-test and Limma package of R/Bioconductor to identify differentially expressed circRNAs, miRNAs and mRNAs between breast cancer tissues and normal breast tissues. Differentially expressed circRNAs were filtered by absolute value of log (fold change (FC)) > 2 and adj. p value < 0.05. Differentially expressed miRNAs were filtered by absolute value of log (fold change (FC)) > 1 and p value < 0.05. Differentially expressed mRNAs were filtered by absolute value of log (fold change (FC)) > 1 and adj. p value < 0.05. Heatmap package of R was used to conduct heatmaps of differentially expressed circRNAs, miRNAs and mRNAs.

### Construction of the circRNA-miRNA-mRNA network

We got information about gene locations, source genes and lengths of circRNAs from circBase website (http://www.circbase.org/). We predicted target miRNAs which may bind to differentially expressed circRNAs using the cancer-specific circRNA database (CSCD) (http://gb.whu.edu.cn/cscd/). We further intersected these target miRNAs with differentially expressed miRNAs in GSE143564. Then we used miRDB and TargetScan databases to predict target mRNAs of the miRNAs intersection. We intersected these target mRNAs with differentially expressed mRNAs in GSE50428. We constructed the circRNA-miRNA-mRNA network according to circRNA-miRNA and miRNA-mRNA correspondence. The expression of miRNAs in the network was opposite to that of circRNAs and mRNAs. We used Cytoscape 3.8.0 to visualize the ceRNA network.

### GO and KEGG pathway enrichment analysis

The potential functions of the differentially expressed circRNAs were further studied by exploring the target mRNAs in the ceRNA network. GO and KEGG pathway enrichment of target mRNAs were analyzed and visualized by using the org.hs.eg.db, clusterProfiler, enrichplot and ggplot2 packages of R/Bioconductor. GO is mainly used to analyze mRNA functions enriched in cell component (CC), molecular function (MF) and biological process (BP). KEGG is used to analyze mRNA functions involved in multiple pathways.

### Survival analysis

To analyze the relationship between mRNAs in the ceRNA network and overall survival of breast cancer patients, we downloaded mRNA expression data and prognostic information from TCGA database. We preformed the overall survival analysis using survival and survminer packages of R/Bioconductor. The mRNAs which were significantly associated with survival time (p < 0.5) were selected to construct a subnetwork associated with prognosis.

### Ethics approval and consent to participate

Our study was approved by the First Affiliated Hospital of Nanjing Medical University Ethical Committee (2021-SR-408). All included patients gave their written informed consent. All methods in our study were carried out in accordance with Declaration of Helsinki. All experimental protocols were approved by the First Affiliated Hospital of Nanjing Medical University Ethical Committee.

## Results

### CircRNA expression profiles of breast cancer and matched paracancer tissues

**Figure 1:**
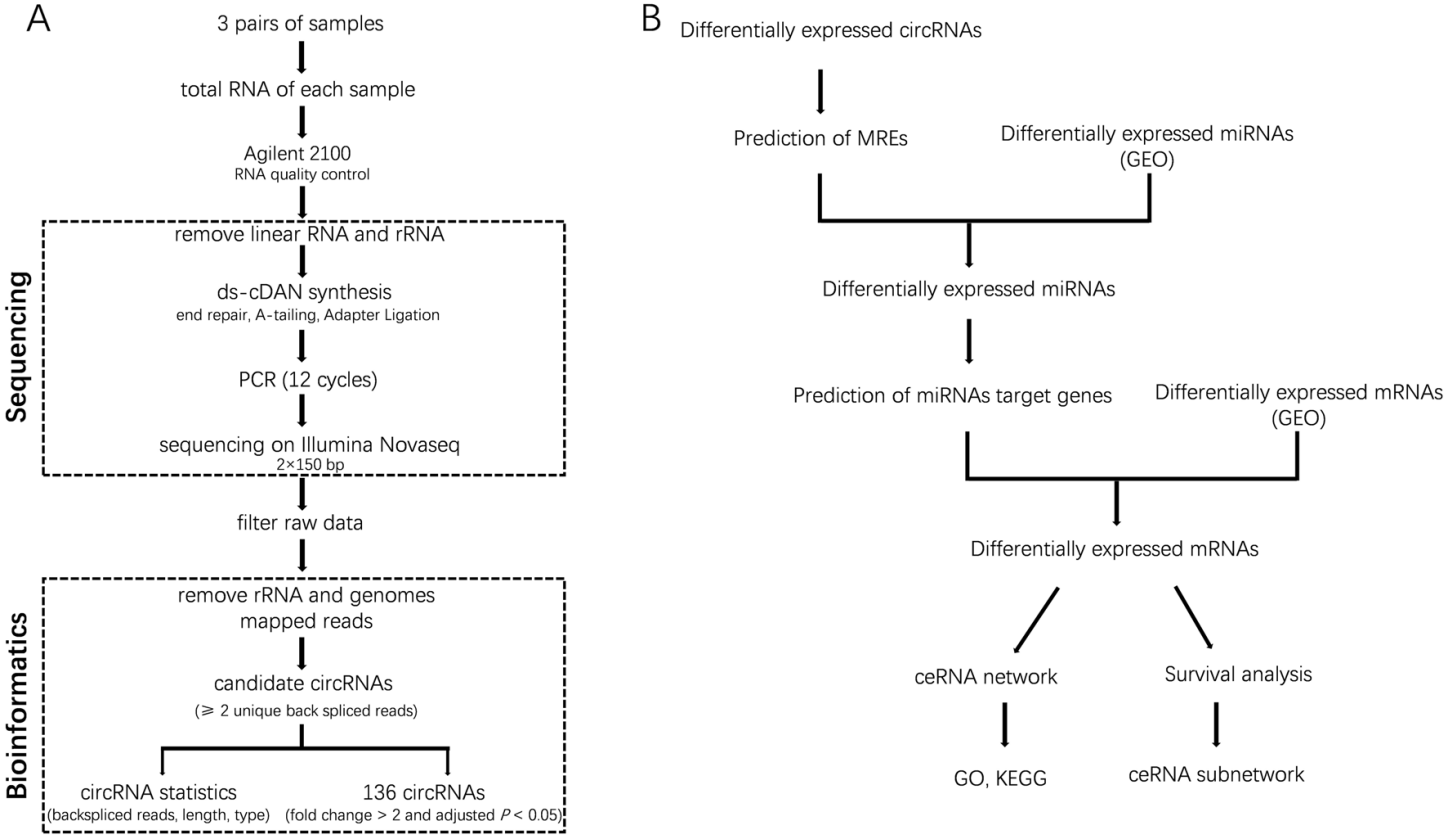
(**A**) CircRNA sequencing library construction method, sequencing protocol and flow chart of bioinformatics analysis. (**B**) Flow chart of ceRNA network construction.

**Figure 2:**
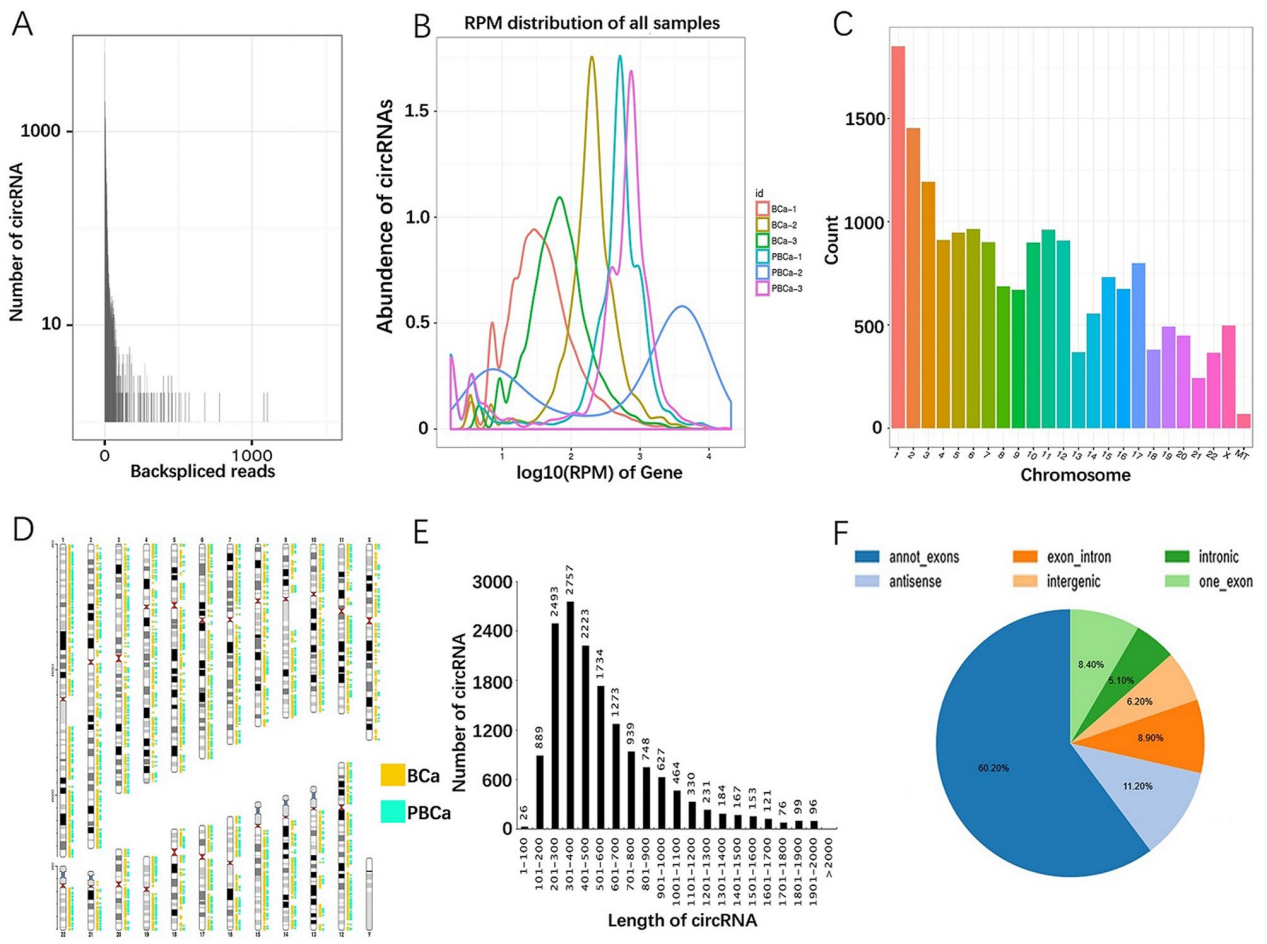
Statistics of identified circRNAs. (**A**) Number and backspliced reads of the circRNAs in 3 paired breast cancer tissues and matched paracancer tissues. (**B**) Horizontal axis: expression levels of circRNAs computed using RPM. Density is the number of genes on the horizontal axis divided by total number of expressed genes. (**C**,**D**) Number and distribution of circRNAs on each chromosome (count: number of circRNAs distributed on each chromosome). (**E**) Length distribution of circRNAs. (**F**) Type distribution of circRNAs. Compositions: Annot_exon: completely exons; Exon_intron: exons and intron; Intronic: intrones; One_exon: one exon. And locations: Antisense: on antisense chains of genes; Intergenic: on intergenic region.

We acquired rRNA and linear-depleted RNA sequencing data of 3 pairs of breast cancer and paracancer tissues. The sequencing process and construction of ceRNA network are shown in Fig. 1. We identified 17,966 circRNA candidates with at least 2 unique backspliced reads (Fig. 2A). RPM distribution of all samples is displayed in Fig. 2B. The number and distribution of circRNAs on each chromosome are shown in Fig. 2C,D. There is no obvious difference in chromosome distribution between cancer tissues and normal tissues. Totally 6591 existing circRNAs in circBase (36.69%) and 11,375 novel circRNAs (63.31%) were identified. The circRNAs are mainly 100 to 1000 nucleotides long (Fig. 2E). About 10,813 circRNAs are annot_exons (60.2%) and other types are evenly distributed: antisense 11.2%, exon_intron 8.9%, intergenic 6.2%, intronic 5.1% and one_exon 8.4% (Fig. 2F). CircRNAs were more highly expressed in breast cancer tissues than in paracancer tissues.

### CircRNA expression data and differentially expressed circRNAs

**Figure 3:**
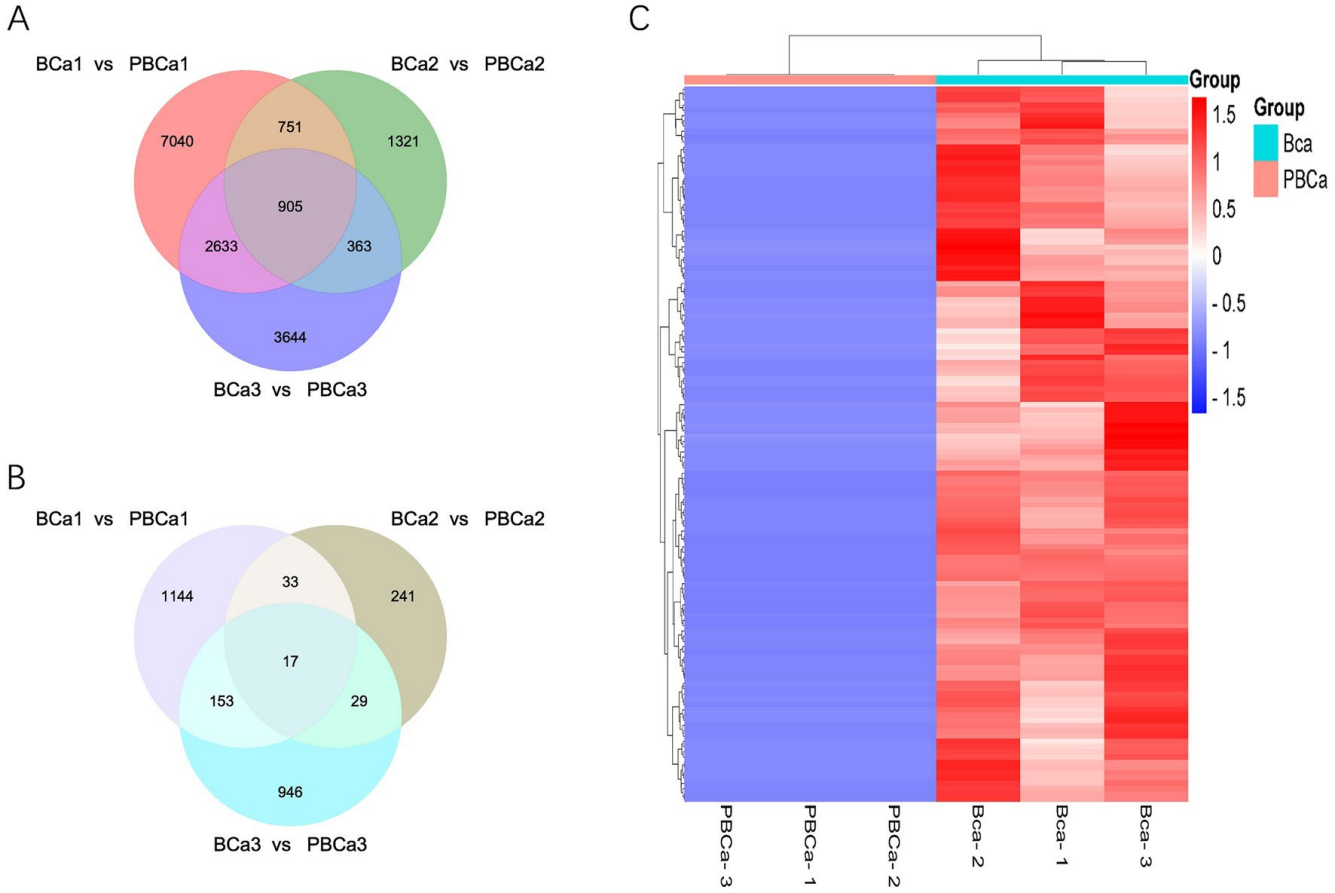
CircRNA expression data and differentially expressed circRNAs. (**A**) Venn diagram showed the number of circRNAs upregulated in breast cancer tissues respectively and their intersections. (**B**) Venn diagram showed the number of circRNAs downregulated in breast cancer tissues respectively and their intersections. (**C**) Heatmap analysis of these circRNAs with fold change > 2 and adjusted P < 0.05 (Y axis represents the expression level of different circRNAs; Cluster analysis was carried out in both X and Y directions).

A total of 12,676 circRNAs were identified in the first pair of samples (11,329 upregulated and 1347 downregulated). 3660 circRNAs were identified in the second pair of samples (3340 upregulated and 320 downregulated). 8690 circRNAs were identified in the third pair of samples (7545 upregulated and 1145 downregulated). The intersections of upregulated and downregulated circRNAs in 3 pairs of samples are shown in Venn diagram (Fig. 3A,B). To acquire potential functional circRNAs associated with breast cancer metastasis, we selected 136 circRNAs with significant differential expression using paired-samples t-test with adjusted *P value* < 0.05 and fold change > 2. These 136 differentially expressed circRNAs are all upregulated in breast cancer tissues and presented in Fig. 3C and Table S1.

### Differentially expressed miRNAs and mRNAs

**Figure 4:**
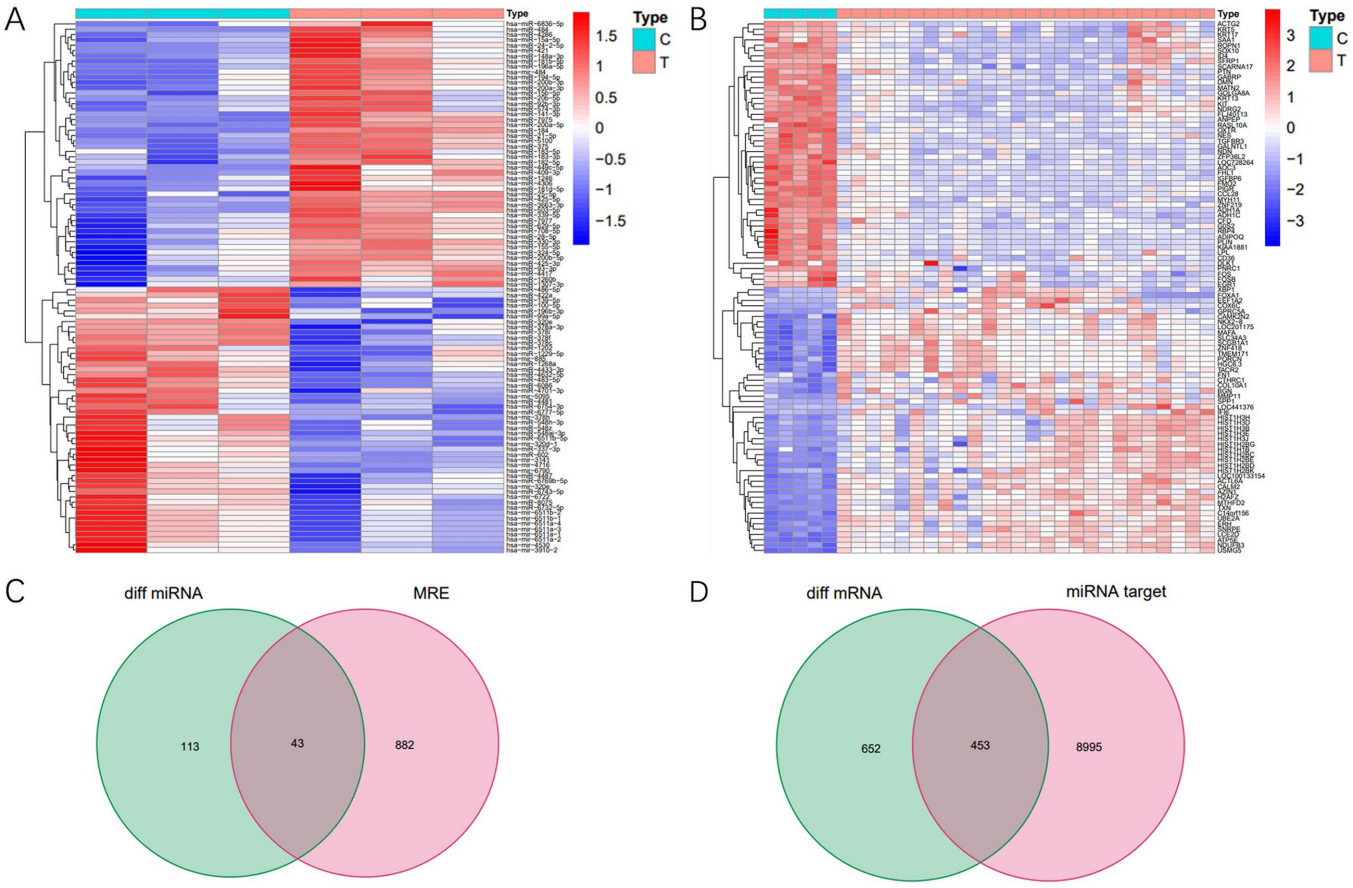
(**A**) Heatmap analysis of top 50 upregulated and downregulated miRNAs from miRNA dataset (GSE143564). (**B**) Heatmap analysis of top 50 upregulated and downregulated mRNAs from mRNA dataset (GSE50428). (**C**) Venn diagram showed intersecting miRNAs of differentially expressed miRNAs and predicted miRNAs. (**D**) Venn diagram showed intersecting mRNAs of differentially expressed mRNAs and target mRNAs.

We identified 156 miRNAs and 1105 mRNAs with differentially expression from miRNA and mRNA datasets (GSE143564 and GSE50428). The top 50 upregulated and downregulated miRNAs and mRNAs are shown in Fig. 4A,B. We predicted that 925 miRNAs might bind to the top 20 differentially expressed circRNAs from CSCD. Then we got 43 intersecting miRNAs of differentially expressed miRNAs and predicted miRNAs (Fig. 4C). We obtained 9448 potential target mRNAs of 43 intersecting miRNAs by miRDB and TargetScan databases. Then we got 453 intersecting mRNAs of differentially expressed mRNAs and target mRNAs (Fig. 4D).

### Construction of a circRNA-miRNA-mRNA network

**Figure 5:**
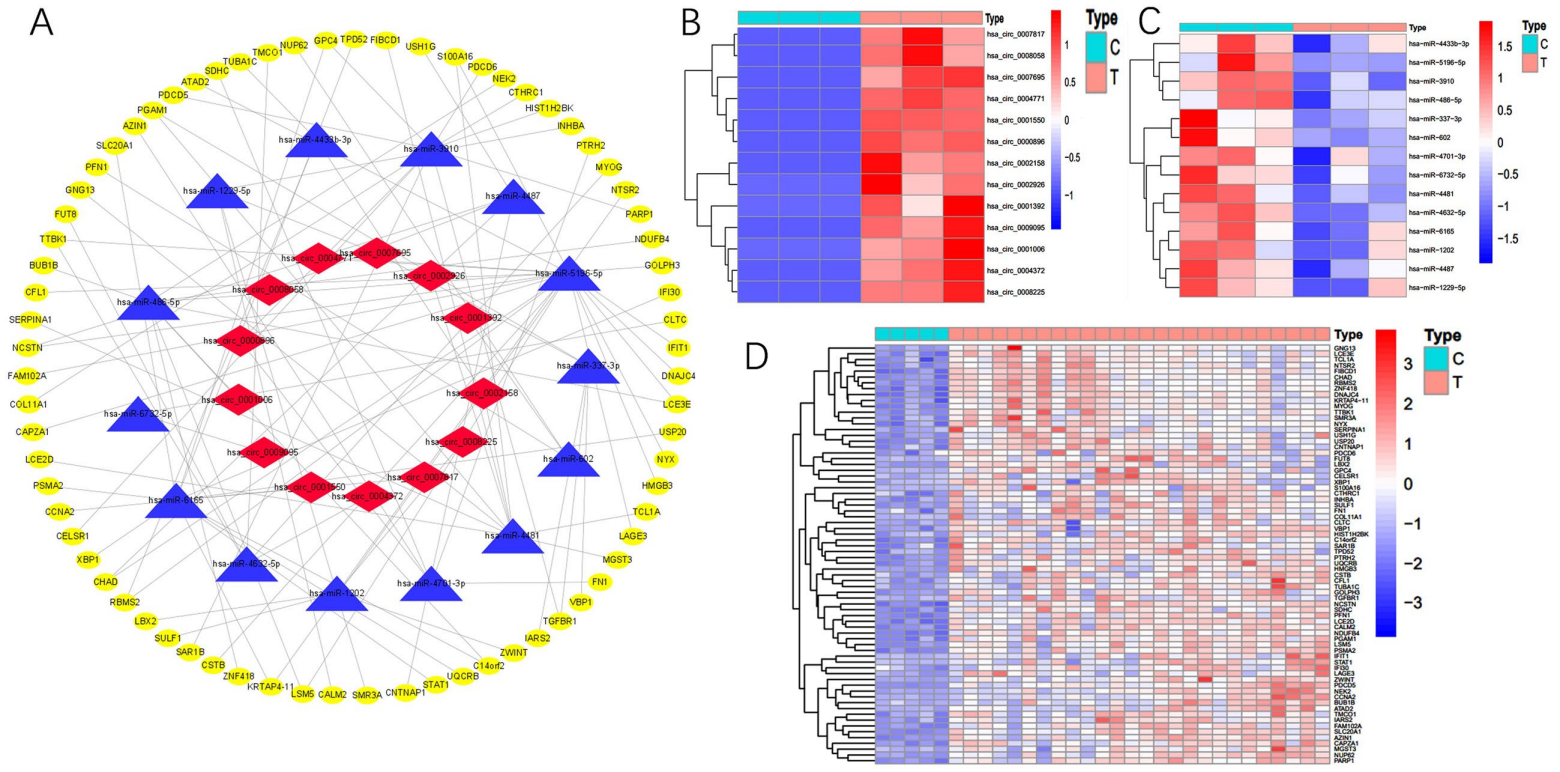
(**A**) CeRNA network of circRNA-miRNA-mRNA interactions in breast cancer. Red indicates circRNAs, blue indicates miRNAs, and yellow indicates mRNAs. (**B**) Expression of the 13 circRNAs in 3 pairs of breast cancer and paracancer tissues. (**C**) Expression of the 14 miRNAs in GSE143564 dataset containing 3 breast cancer tissues and 3 adjacent tissues. (**D**) Expression of the 72 mRNAs in GSE50428 dataset containing 5 normal breast tissues and 26 breast cancer tissues.

We constructed the network using 20 circRNAs, 43miRNAs and 453 mRNAs and leave out the RNAs which can’t form a complete circRNA-miRNA-mRNA axis. Finally, there are 13 circRNAs, 14 miRNAs and 72 mRNAs in this network (Fig. 5A) and the expression of these RNAs were shown if Fig. 5B–D.

### GO and KEGG analysis

**Figure 6:**
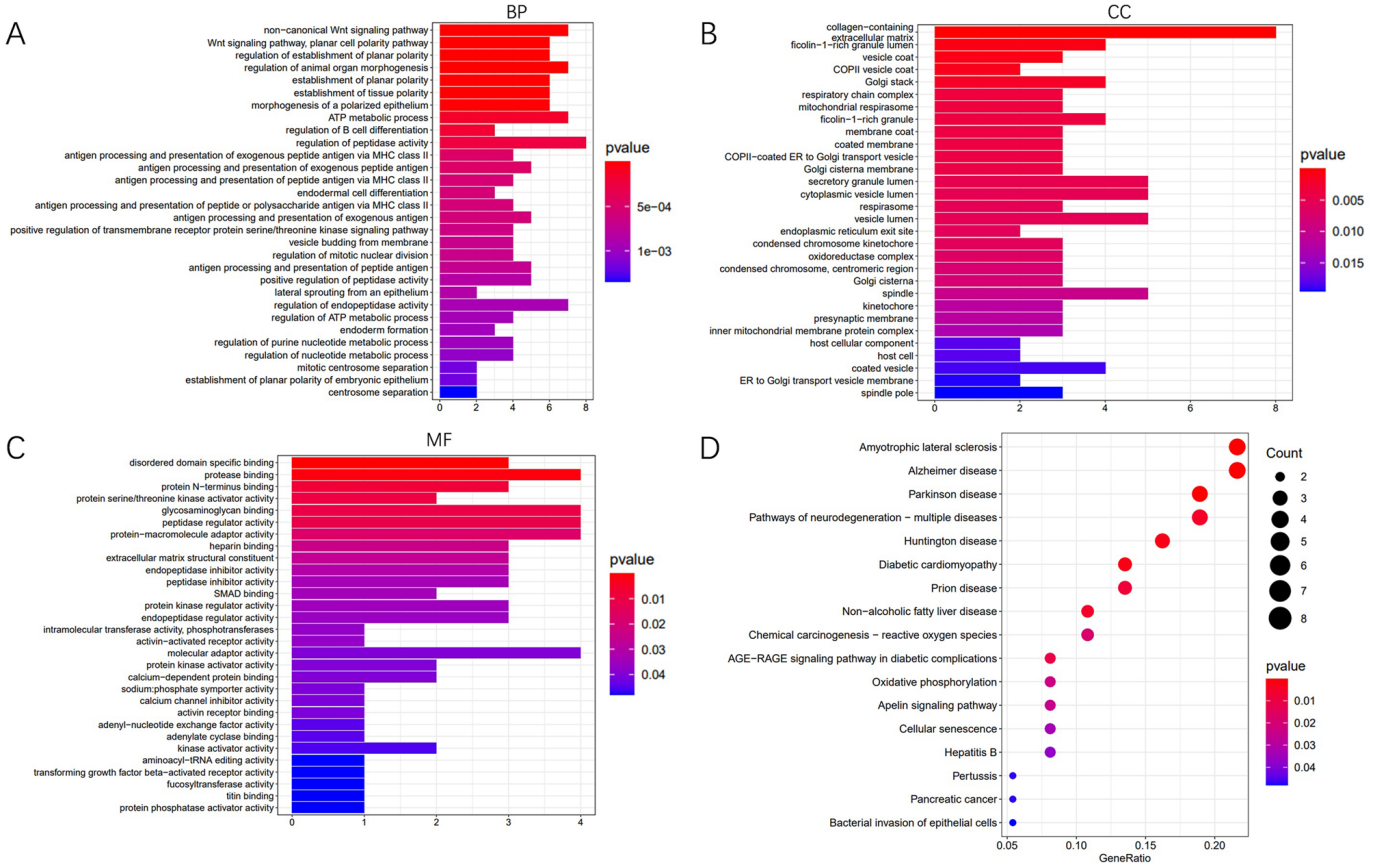
GO and KEGG enrichment analyses of the 72 mRNAs in the ceRNA network: (**A**) biological process, (**B**) cellular component and (**C**) molecular function. (**D**) Enrichment analysis of KEGG pathways^20–22^.

The potential functions of the 13 selected circRNAs were further studied by exploring the 72 mRNAs through GO annotation and KEGG pathway enrichment analyses. The top 30 enriched GO terms of each class are illustrated in Fig. 6A–C. The mRNAs were partly enriched in ‘non-canonical Wnt signaling pathway’, ‘transforming growth factor beta-activated receptor activity’ and ‘protein kinase regulator activity’. The KEGG pathway analysis is illustrated in Fig. 6D. The mRNAs were partly enriched in ‘Chemical carcinogenesis—reactive oxygen species’, ‘Oxidative phosphorylation’ and ‘Cellular senescence’. It has been reported that oxidative phosphorylation is involved in drug resistance and metastasis of breast cancer^16,17^. Alteration of the cellular senescence pathway through multiple molecular mechanisms may play an important role in the progression of breast cancer^18,19^.

### Survival analysis and construction of survival-related subnetwork

**Figure 7:**
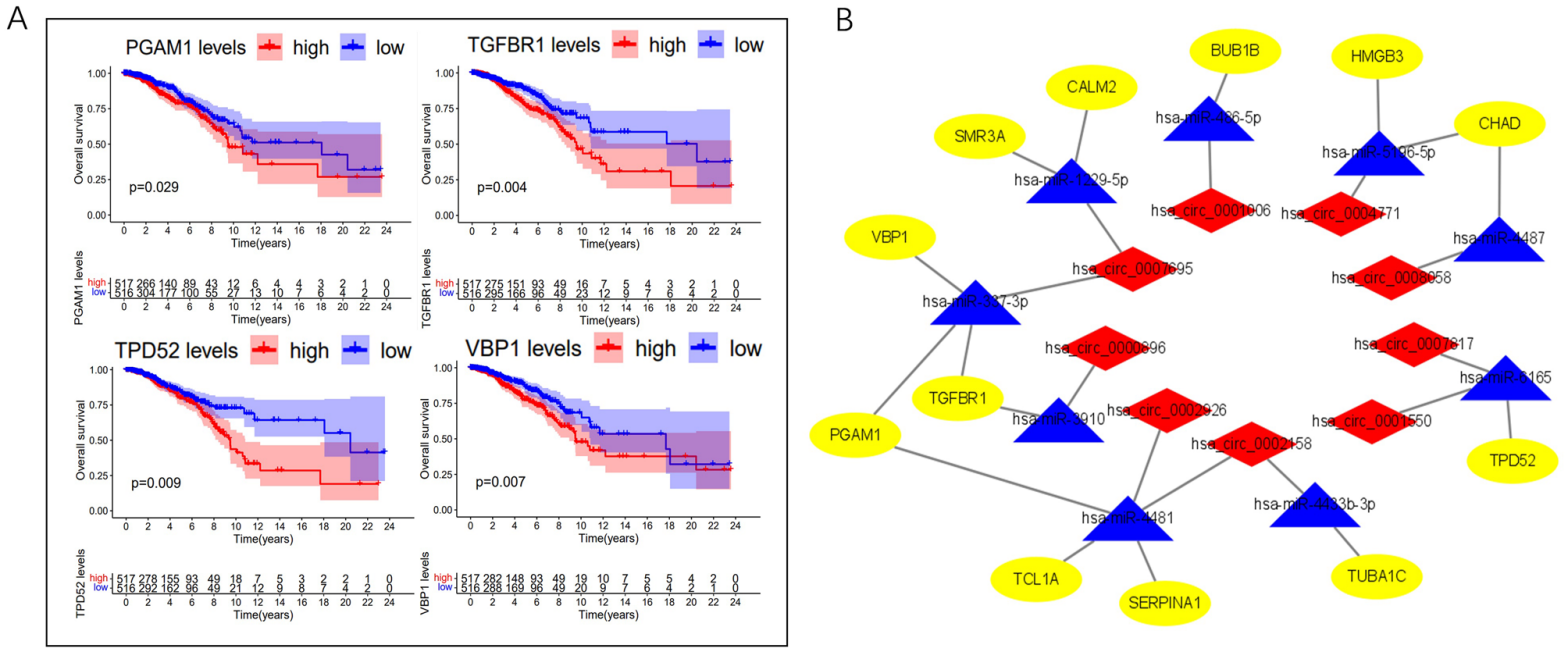
TCGA database was used to analyse survival prognosis on the 72 mRNAs in the ceRNA network. (**A**) The PAAM1, TGFBR1, TPD52 and VBP1 gene high expression groups in breast cancer samples had worse prognosis (P < 0.05). (**B**) Construction of prognostic ceRNA subnetwork with these survival-associated mRNAs.

We downloaded mRNA expression data of 1053 breast cancer samples and prognostic information from TCGA database. We conducted survival analysis on the 72 mRNAs in the ceRNA network. There were 12 mRNAs significantly associated with prognosis in breast cancer patients (partly displayed in Fig. 7A). Prognostic ceRNA subnetwork was constructed with these survival-associated mRNAs (Fig. 7B).

## Discussion

Breast cancer patients with distant metastasis and LNM are faced with worse survival prognosis. Therefore, our study focuses on metastatic breast cancer and explores the detailed mechanisms. Misregulation of transcriptome contributes to multiple diseases, including cancers^23^. Despite many studies on linear transcriptomes, accumulating evidence indicates circRNAs participate in cancer progression. Many circRNAs have been demonstrated by the advancement of high-throughput RNA sequencing to be dysregulated in cancers, such as gastric cancer, bladder cancer and hepatocellular cancer^24–26^.

Herein, we acquired the circRNA expressions of breast cancer tissues with LNM and of matched paracancer tissues using RNA sequencing. We identified 17,966 circRNA candidates, including 6591 existing circRNAs and 11,375 novel circRNAs. These novel circRNAs are of particular interest for further study. The total expression of circRNAs in breast cancer tissues was upregulated. Reportedly, however, total expression of circRNAs was downregulated in glioblastoma compared with normal brain tissues^12^. Most circRNAs are entirely composed of exons. Then we acquired 136 significantly differential expressed circRNAs using paired-samples t-test. We note that the significantly upregulated circRNAs are much more than the downregulated ones in breast cancer tissues compared with normal breast tissues in this study.

To explore the role of circRNAs in breast cancer, we obtained the miRNA and mRNA expression profiles from GEO and identified the miRNAs and mRNAs with differential expressions. Then we constructed a circRNA-mediated ceRNA network. we analyzed the mRNAs in the netwrok by performing GO and KEGG pathway enrichment tests. Results showed the mRNAs were involved in some cancer-related GO terms, such as ‘non-canonical Wnt signaling pathway’ and ‘transforming growth factor beta-activated receptor activity’. Some evidence shows that cancer cells acquire metastatic and migratory capacity through hijacking the non-canonical Wnt signaling pathway. For example, melanoma and gastric cancer cells acquired increased migration and metastasis while Wnt5a was overexpressed^27,28^. Non-canonical Wnt/ Ca^2+^ signaling pathway also plays a role in cisplatin resistance of ovarian cancer^29^. These results demonstrate that circRNAs in this network might play a critical role in progression of breast cancer.

To further explore the role of circRNAs in breast cancer prognosis, we carried out survival analysis on the 72 mRNAs in the ceRNA network based on TCGA database. We found that 12 mRNAs were associated with prognosis of breast cancer. Then we constructed a survival-associated ceRNA subnetwork. There were 9 circRNAs, 9miRNAs and 12 mRNAs in the network.

## Conclusions

we showed circRNA expression profile of breast cancer tissues and identified 136 circRNAs differentially expressed between breast cancer and paracancer tissues. We aslo identified 156 miRNAs and 1105 mRNAs with differentially expression from public database. Then we constructed a circRNAs-miRNA-mRNA network. 12 mRNAs were associated with prognosis of breast cancer based on TCGA database. We also constructed a circRNAs-mediated subnetwork which might be related to prognosis of breast cancer. The results in this study provide a feasible molecular mechanism of metastasis of breast cancer. Further studies revealing the functions of these circRNAs, which are involved in breast cancer invasion and metastasis, will provide a new perspective for treatment of metastatic breast cancer.

## Supplementary Information

Supplementary Table S1.

## Data Availability

The circRNA sequences data reported in this study was archived in the Sequence Read Archive (SRA) with the accession number PRJNA822662. Data could be available at: https://www.ncbi.nlm.nih.gov/bioproject/PRJNA822662.

## References

[1] Sung H, Ferlay J, Siegel RL (2021). Global cancer statistics 2020: GLOBOCAN estimates of incidence and mortality worldwide for 36 cancers in 185 countries. CA Cancer J. Clin..

[2] Berry DA (2005). Effect of screening and adjuvant therapy on mortality from breast cancer. N. Engl. J. Med..

[3] Munoz, D. *et al.* Effects of screening and systemic adjuvant therapy on ER-specific US breast cancer mortality. *J. Natl. Cancer Inst.* **106**(11), dju289. 10.1093/jnci/dju289 (2014). 10.1093/jnci/dju289 PMC4271026 25255803

[4] DeSantis CE (2017). Breast cancer statistics, 2017, racial disparity in mortality by state. CA Cancer J. Clin..

[5] Qu S (2015). Circular RNA: A new star of noncoding RNAs. Cancer Lett..

[6] Barrett SP, Salzman J (2016). Circular RNAs: Analysis, expression and potential functions. Development.

[7] Li X, Yang L, Chen LL (2018). The biogenesis, functions, and challenges of circular RNAs. Mol. Cell.

[8] He R (2017). circGFRA1 and GFRA1 act as ceRNAs in triple negative breast cancer by regulating miR-34a. J. Exp. Clin. Cancer Res..

[9] Wu J (2018). CircIRAK3 sponges miR-3607 to facilitate breast cancer metastasis. Cancer Lett..

[10] Zhou J (2018). Downregulation of hsa_circ_0011946 suppresses the migration and invasion of the breast cancer cell line MCF-7 by targeting RFC3. Cancer Manag. Res..

[11] Liu Y (2018). Circular RNA-MTO1 suppresses breast cancer cell viability and reverses monastrol resistance through regulating the TRAF4/Eg5 axis. Int. J. Oncol..

[12] Yang Y (2018). Novel role of FBXW7 circular RNA in repressing glioma tumorigenesis. J. Natl. Cancer Inst..

[13] Langmead B, Salzberg SL (2012). Fast gapped-read alignment with Bowtie 2. Nat. Methods.

[14] Kim D (2013). TopHat2: Accurate alignment of transcriptomes in the presence of insertions, deletions and gene fusions. Genome Biol..

[15] Memczak S (2013). Circular RNAs are a large class of animal RNAs with regulatory potency. Nature.

[16] Evans KW (2021). Oxidative phosphorylation is a metabolic vulnerability in chemotherapy-resistant triple-negative breast cancer. Cancer Res..

[17] Ramchandani D (2021). Copper depletion modulates mitochondrial oxidative phosphorylation to impair triple negative breast cancer metastasis. Nat. Commun..

[18] Yang D (2020). Wogonin induces cellular senescence in breast cancer via suppressing TXNRD2 expression. Arch. Toxicol..

[19] Sossey-Alaoui K (2019). The Kindlin2-p53-SerpinB2 signaling axis is required for cellular senescence in breast cancer. Cell Death Dis..

[20] Kanehisa M (2021). KEGG: Integrating viruses and cellular organisms. Nucleic Acids Res..

[21] Kanehisa M (2019). Toward understanding the origin and evolution of cellular organisms. Protein Sci..

[22] Kanehisa M, Goto S (2000). KEGG: Kyoto encyclopedia of genes and genomes. Nucleic Acids Res..

[23] Lee TI, Young RA (2013). Transcriptional regulation and its misregulation in disease. Cell.

[24] Xie F (2018). Circular RNA BCRC-3 suppresses bladder cancer proliferation through miR-182-5p/p27 axis. Mol. Cancer.

[25] Meng J (2018). Twist1 regulates vimentin through Cul2 circular RNA to promote EMT in hepatocellular carcinoma. Cancer Res..

[26] Liu H (2018). Circular RNA YAP1 inhibits the proliferation and invasion of gastric cancer cells by regulating the miR-367-5p/p27 (Kip1) axis. Mol. Cancer.

[27] Kurayoshi M (2006). Expression of Wnt-5a is correlated with aggressiveness of gastric cancer by stimulating cell migration and invasion. Cancer Res..

[28] Weeraratna AT (2002). Wnt5a signaling directly affects cell motility and invasion of metastatic melanoma. Cancer Cell.

[29] Huang L (2016). Role of Wnt/β-catenin, Wnt/c-Jun N-terminal kinase and Wnt/Ca(2+) pathways in cisplatin-induced chemoresistance in ovarian cancer. Exp. Ther. Med..

